# Delay-Induced Multistability and Loop Formation in Neuronal Networks with Spike-Timing-Dependent Plasticity

**DOI:** 10.1038/s41598-018-30565-9

**Published:** 2018-08-13

**Authors:** Mojtaba Madadi Asl, Alireza Valizadeh, Peter A. Tass

**Affiliations:** 1 Department of Physics, Institute for Advanced Studies in Basic Sciences (IASBS), Zanjan, 45195-1159 Iran; 2 School of Cognitive Sciences, Institute for Research in Fundamental Sciences (IPM), Tehran, 19395-5746 Iran; 3 Department of Neurosurgery, School of Medicine, Stanford University, Stanford, CA 94305 USA

## Abstract

Spike-timing-dependent plasticity (STDP) adjusts synaptic strengths according to the precise timing of pre- and postsynaptic spike pairs. Theoretical and computational studies have revealed that STDP may contribute to the emergence of a variety of structural and dynamical states in plastic neuronal populations. In this manuscript, we show that by incorporating dendritic and axonal propagation delays in recurrent networks of oscillatory neurons, the asymptotic connectivity displays multistability, where different structures emerge depending on the initial distribution of the synaptic strengths. In particular, we show that the standard deviation of the initial distribution of synaptic weights, besides its mean, determines the main properties of the emergent structural connectivity such as the mean final synaptic weight, the number of two-neuron loops and the symmetry of the final structure. We also show that the firing rates of the neurons affect the evolution of the network, and a more symmetric configuration of the synapses emerges at higher firing rates. We justify the network results based on a two-neuron framework and show how the results translate to large recurrent networks.

## Introduction

Spike-timing-dependent plasticity (STDP)^1–5^ modifies synaptic strengths according to the relative timing of pre- and postsynaptic spike pairs. When the postsynaptic spike follows the presynaptic spike, a potentiation of the corresponding synaptic strength is induced by the STDP mechanism, whereas the synaptic strength is depressed in the opposite case^2^. STDP is a local learning rule, where the synaptic modification only depends on the joint pre-post activity of the corresponding synapse. However, STDP may significantly impact on the global dynamics of neuronal networks, so that qualitatively different connectivity patterns can emerge under the influence of STDP^6–17^. It is commonly accepted that the temporally asymmetric learning window of the classic pair-based STDP eliminates strong bidirectional loops between neuronal connections^18–23^. Although this interesting feature of STDP can explain the emergence of feedforward networks^4,19,24,25^, it is in contradiction to the prevalence of recurrent connections in cortical circuits^26–30^.

However, it is possible to stabilize strong bidirectional connections by employing variations of STDP^22^ or by considering independent noise^31^ in the plastic neuronal networks. On the other hand, as shown recently, by taking into account dendritic and axonal propagation delays, the conventional pair-based additive STDP may lead to the emergence of different connectivity patterns including both unidirectional and bidirectional connections, or decoupled neurons^32^.

An asymmetric learning window of STDP profile can push the network dynamics toward a synchronized state with strongly up-regulated synaptic connections, or conversely, the network can evolve into a desynchronized regime with down-regulated synaptic strengths^33–37^. It was shown that the initial mean synaptic coupling determines the finally evolving mean strength of the synaptic connections. Strong initial coupling leads to synchronized dynamics that can be enhanced and preserved by STDP, while weak initial connections lead to desynchronized states, i.e., there is a multistability of synchronized and desynchronized states in the plastic neuronal networks^33,34,38^. Hence, STDP-driven neuronal populations can induce different multistable attractor states characterized by specific coupling regimes^33,34,38^.

Dendritic and axonal propagation delays within and between brain areas may assume different values and need not be identical. Dendritic delays are typically smaller than axonal delays^20^. However, dendritic delays may range from sub-millisecond to a few milliseconds^39,40^, e.g. Agmon-Snir and Segev^39^ demonstrated that the dendritic delay in octopus cells is about 0.5 ms. Axonal propagation delays may range from 0.3 ms in thalamo-cortical connections^41^ and 1 ms in cortico-tectal connections^42^ to 20 ms in cortico-cortical connections^43^, and even more, up to 40 ms in cortico-thalamic circuits^42^.

The presence of dendritic and axonal propagation delays can regulate the emergent structures of STDP-driven plastic neuronal populations^20,32^. Propagation delays affect the function of STDP in two different ways. First, the total propagation delay *τ* = *τ*_d_ + *τ*_a_, i.e., the sum of the dendritic *τ*_d_ and axonal *τ*_a_ propagation delays, determine the synchronization tendency of the coupled neurons and the time difference of spiking of the neurons^44–50^. Second, since the effect of pre- and postsynaptic neurons arrive at the synapse after axonal and dendritic delays, respectively, the difference of the two propagation delays *ξ* = *τ*_d_ − *τ*_a_, changes the relative timing at the synapse. We recently showed^32^ that by incorporating propagation delays in a plastic neuronal network model, bidirectional connections can be retained and potentiated despite the previously reported simulation results with conventional STDP in the absence of delays^33,34,38^.

In this study, we focus on the multistability of the structural dynamics of the network with respect to the distribution of the initial synaptic weights in coupled networks with delays. Multistability was previously reported with respect to the mean initial synaptic weight: The final mean synaptic strength mainly depends on the initial average weight^34,38^. Here we show that within the present framework not only the mean, but also the standard deviation of the initial connections crucially determines the final mean strength of the synapses. Moreover, the presence of bidirectional connections and the symmetry of the final structure is determined by the initial discrepancy of the synaptic weights. We show that the multistability of the network can be explained in a modular way, by the presence of different attractors for a two-neuron motif in the two-dimensional space of the initial connection strengths. We also show that the domain of attraction of each final structural state depends on the firing frequency of the neurons: At high firing rates the attraction domain for symmetric connections grows in expense of shrinking that of the unidirectional connections. This leads to a more symmetric final structure when the neurons are firing at higher rates. Intriguingly, all of these nontrivial phenomena are only seen when the propagation delays are incorporated in the formulation of the model: Without propagation delays, any initial preparation ends up with unidirectional connections regardless of the firing rate of the neurons and the initial weights of the synapses.

## Results

### Theoretical Framework

We consider two neuronal oscillators described in a phase reduced model. In this approach, the evolution equation for the relative phase of the two neuronal oscillators can be written as follows (for the derivation see Methods, Eq. (6)):

1 $$\dot{\chi }={\rm{\Omega }}+\frac{1}{2\pi }({\rm{\Gamma }}\,\tan \,\psi \,\cot \,\chi -1),\,{\rm{\Gamma }}=|\frac{{g}_{12}-{g}_{21}}{{g}_{12}+{g}_{21}}|,$$

where *χ* = *φ*_2_ − *φ*_1_ is the phase lag, ${\rm{\Omega }}\,=\,2\pi {\rm{\Delta }}\nu$ that Δ*ν* = *ν*_2_ − *ν*_1_ is the frequency mismatch, and *ψ* = 2*πντ* is the rescaled delay with *τ* = *τ*_d_ + *τ*_a_ being the sum of dendritic *τ*_d_ and axonal *τ*_a_ propagation delays. The values for dendritic and axonal propagation delays are chosen based on the experimental observations in cortical areas^42,43^. The quantity Γ reflects the relative difference of the synaptic strengths in two opposite directions, where *g*_*ij*_ with *i*, *j* = 1, 2 (*j* ≠ *i*) is the synaptic strength of the synapse connecting presynaptic neuron *j* to postsynaptic neuron *i*.

The synapses connecting the two neurons are subjected to classic pair-based STDP rule where the synaptic strengths *g*_*ij*_ = *g*_*ij*_(*t*) are modified based on the delayed time lag between pre- and postsynaptic activity at synaptic site, i.e., ${\rm{\Delta }}{t^{\prime} }_{ij}={\rm{\Delta }}{t}_{ij}+\xi$^32^ where Δ*t*_*ij*_ = *t*_*i*_ − *t*_*j*_ is the time lag in the absence of propagation delays and *ξ* = *τ*_d_ − *τ*_a_ is the difference between dendritic and axonal propagation delays. The synaptic strengths are updated by an additive rule at each step *g*_*ij*_ → *g*_*ij*_ + Δ*g*_*ij*_, according to the following learning function (see Methods):

2 $${\rm{\Delta }}{g}_{ij}={A}_{\pm }\,{\rm{sgn}}({\rm{\Delta }}{t^{\prime} }_{ij})\,\exp (\,-\,|{\rm{\Delta }}{t^{\prime} }_{ij}|/{\tau }_{\pm }),$$

where *A*_+_(*A*_−_) and *τ*_+_(*τ*_−_) are the learning rate and the effective time window of synaptic potentiation (depression), respectively, and sgn(Δ*t*) is the sign function. The synaptic strengths are bounded in the range (*g*_min_, *g*_max_) = [0.05, 1] by the hard bound saturation constraint^4,51^: The synaptic strengths are set to *g*_min_ (*g*_max_) once they cross the lower (upper) limit of their allowed range. We consider a balanced STDP profile^4,19^ with equal potentiation and depression contribution, i.e. *A*_+_ = *A*_−_ and *τ*_+_ = *τ*_−_, in order to extract the pure influence of propagation delay and oscillation frequency on the synaptic dynamics. We assumed that the changes in synaptic strengths are slow in comparison to the fast timescale of the system, therefore, the neurons remain phase-locked despite of eventual change in the synaptic strengths. Given the firing frequency *ν* and assuming that the frequency mismatch between the two oscillators is negligible, i.e. ${\rm{\Omega }}\simeq 0$, the fast timescale equation of the two-neuron motif (Eq. (1)) exhibits a stable fixed point which is given by Eq. (9), ${\chi }^{\ast }={\rm{t}}{\rm{a}}{{\rm{n}}}^{-1}({\rm{\Gamma }}\,{\rm{\tan }}\,\psi )$, and hence firing time difference of two neurons at the stable state will be Δt^*^ = χ^*^/(2*πν*). These values could be inserted in Eq. (3) to give the evolution of the synaptic connections^32,52^.

### Bistability in the Two-Neuron Motif

**Figure 1:**
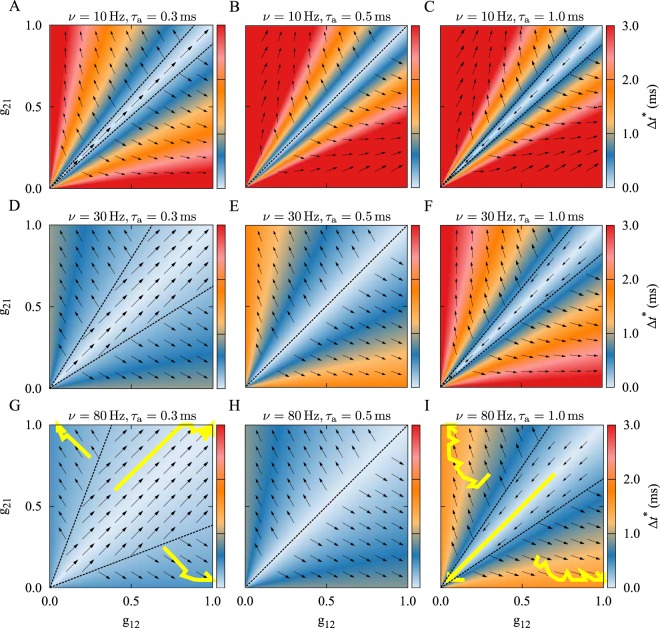
Theoretical prediction of synaptic modification as a function of the initial synaptic strengths in the absence of a frequency mismatch. The colors show the stable fixed point of the time lag Δt^*^ = χ^*^/(2*πν*) of spiking of the neurons given by Eq. (9) and the vector field shows the direction of the change of the synaptic strengths from Eq. (3). The yellow solid curves denote the simulated synaptic evolution for three different initial values. In each row, the firing frequency *ν* is constant, but it increases from top to bottom in each column. Based on the initial synaptic coupling, different attractors may be achieved: Bidirectional/unidirectional (left column), unidirectional (middle column), and decoupled/unidirectional (right column) state. The dendritic propagation delay is ${\tau }_{{\rm{d}}}=0.5\,\,{\rm{ms}}$ and the axonal delay is denoted above each panel. STDP parameters are *A*_+_ = *A*_−_ = 0.005 and *τ*_+_ = *τ*_−_ = 20 ms.

**Figure 2:**
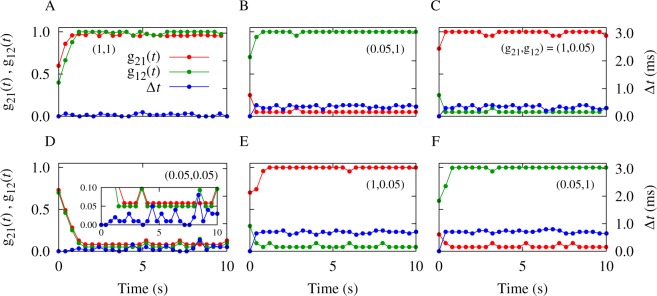
Time course of simulated synaptic strengths and spiking time lag. (**A–C**) Correspond to Fig. 1G with *ν* = 80 Hz and *τ*_a_ = 0.3 ms. (**D–F**) Correspond to Fig. 1I with *ν* = 80 Hz and *τ*_a_ = 1.0 ms. The initially given two-dimensional synaptic strength vector is (*g*_21_(0), *g*_12_(0)) = (0.6, 0.4), (0.2, 0.7), (0.8, 0.2), (0.7, 0.7), (0.7, 0.3), (0.2, 0.6) for (**A**–**F**), respectively. The finally achieved two-dimensional synaptic strength vector (*g*_21_, *g*_12_) is denoted in the figure.

In Fig. 1 we show the synaptic dynamics (illustrated by the vector field) overlaid on the the representation of the instantaneous time lag of the spiking neurons (shown by colors), for the different values of the initial synaptic strengths (*g*_21_(0), *g*_12_(0)). It can be seen that the basin of attraction for the three different final connectivity patterns (bidirectional, unidirectional and decoupled) is determined by the firing frequency *ν* and the difference between dendritic and axonal delays *ξ*. The results shown in Fig. 1 denote the theoretical prediction of different stable structural states that coexist for different combinations of delay and firing frequency. The colors indicate the stable fixed point of the time lag Δt^*^ given by Eq. (9), and the vector field shows the changes of the synaptic strengths from Eq. (3). Given the initial values of the synaptic strengths, the instantaneous color-coded fixed point of the time lag determines the synaptic changes depicted by the vector field, and the subsequent values of the synaptic strengths. The corresponding trajectories of the time evolution of the synaptic strengths resulted from numerical experiments with three different initial values are shown by yellow solid curves in Fig. 1G and I. The numerical solutions practically follow the vector field directions predicted by the analytical results. The time course of the simulated synaptic strengths and the firing time lag are presented in Fig. 2.

Figure 1, left and right columns illustrate that according to different initial arrangements of the connections, the two-neuron motif will end up with one of the bistable coupling regimes: Bidirectional/unidirectional coupling (Fig. 1, left column, dendritic delay is greater), or decoupled/unidirectional coupling (Fig. 1, right column, axonal delay is greater). The basin of attraction of the symmetric coupling regimes (i.e. bidirectional or decoupled final configuration) grows with an increase of the firing frequency (Fig. 1, left and right columns, top to bottom), making it more probable that in the high-frequency firing regime the neurons are either bidirectionally coupled (Fig. 1G) or remain decoupled (Fig. 1I).

**Figure 3:**
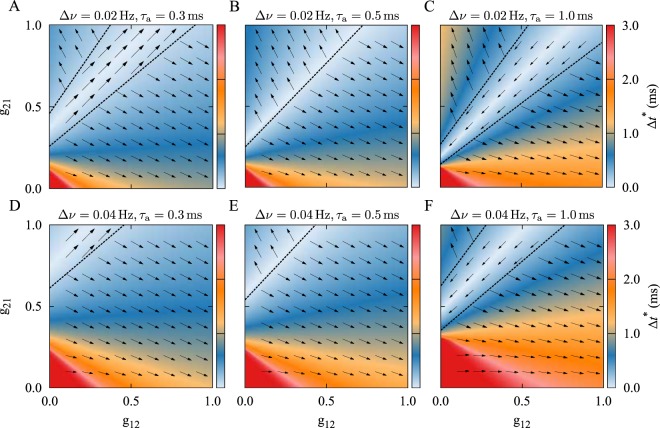
Theoretical prediction of synaptic modification based on the initial synaptic strengths in the presence of a frequency mismatch. In the presence of a frequency mismatch Δ*ν*, the instantaneous fixed point of the time lag is numerically calculated by self-consistently solving Eq. (8) with the Newton-Raphson root-finding method. (**A–C**) Frequency mismatch Δ*ν* = 0.02 Hz with *ν*_2_ > *ν*_1_. (**D–F**) Frequency mismatch Δ*ν* = 0.04 Hz with *ν*_2_ > *ν*_1_. In the presence of frequency mismatch the output synapse of the neuron with higher frequency is more likely to be potentiated, while the reverse synapse is depressed.

We also examined the effect of a frequency mismatch Δ*ν* = *ν*_2_ − *ν*_1_, i.e. the difference between the frequencies of the two oscillators introduced in Eq. (1), on the evolution of the synapses. Expectedly, the frequency mismatch shifts the border of the basins of attraction in a way that the neuron with the greater firing rate more likely wins the competition in generating a stronger outgoing synapse and suppressing the reverse one (see Fig. 3), which is in accordance with previous studies^21,53^. However, for small frequency mismatches (e.g. Δ*ν* = 0.02 Hz in Fig. 3A–C) bistability can still be present, and all three final configurations may be achieved depending on other parameters, i.e., the difference in propagation delays and firing frequencies.

The relation between dendritic and axonal delays is crucial to the emergence of bistability. When dendritic and axonal delays are equal, *ξ* = 0, which is equivalent to ignoring both in the STDP implementation, no bistability emerges and the final connectivity pattern is unidirectional, regardless of the initial synaptic strengths (see Figs 1 and 3, middle columns). This is consistent with the results of Babadi and Abbott^23^, obtained for a balanced STDP profile. Note, in our model the bistability and the possibility of the emergence of symmetric connectivity patterns are observed for symmetric STDP just because the propagation delays are explicitly considered (also see Madadi Asl *et al*.^32^).

**Figure 4:**
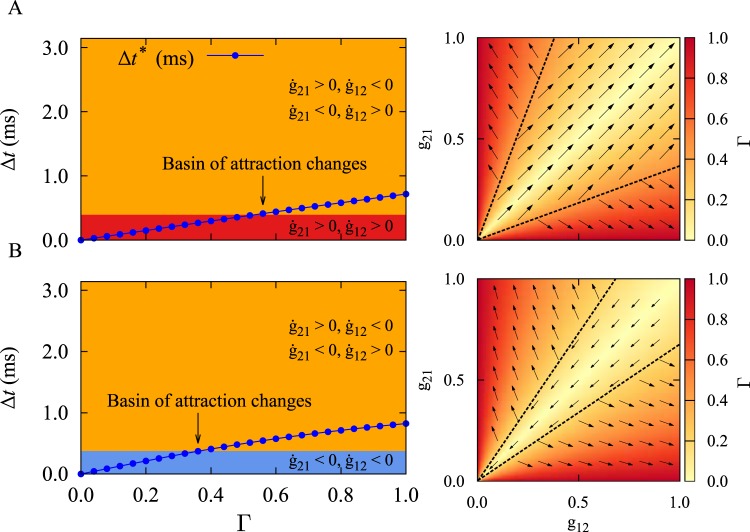
Dependence of the two-neuron results on the relative synaptic strength. (Left panels) The interplay between fixed point of the time lag ${\rm{\Delta }}{t}^{\ast }$ and relative synaptic strength Γ determines the final stable connectivity pattern. Colors show the type of the final synaptic connectivity pattern: Bidirectional (red), unidirectional (orange), and decoupled (blue). The blue curve denotes Δt^*^ calculated from Eq. (9). (**A**) *ν* = 80 Hz and *τ*_a_ = 0.3 ms. (**B**) *ν* = 80 Hz and *τ*_a_ = 1.0 ms. The dendritic propagation delay is *τ*_d_ = 0.5 ms. (Right panels) The color-coded relative synaptic strength Γ is shown based on the synaptic strengths from Eq. (1). The vector field is the same as in Fig. 1.

The dependence of the final stable coupling regimes on the initial values of the synaptic strengths can be explained based on Eq. (9), ${\rm{\Delta }}{t}^{\ast }={\tan }^{-1}({\rm{\Gamma }}\,\tan \,\psi )\mathrm{/(2}\pi \nu )$, that gives the asymptotic time lag between pre- and postsynaptic spikes. Therefore, the instantaneous fixed point of the time lag (Eq. (9)) is determined by the relative synaptic strength Γ, the firing frequency of the oscillation *ν*, and the total delay *τ*. In turn, the instantaneous value of Δt^*^ determines the evolution of the synaptic strengths according to Eq. (3). More specifically, the values of the synaptic strengths determine the relative synaptic strength Γ = |(*g*_12_ − *g*_21_)/(*g*_12_ + *g*_21_)| from Eq. (1), where the interaction of the relative synaptic strength with the fixed point of the time lag ${\rm{\Delta }}{t}^{\ast }$ ultimately determines the final stable coupling regime. The right panels of Fig. 4, show that the color-coded relative synaptic strength Γ is close to zero around the diagonal line (light color) and maximal at the corners (dark color). As shown in Fig. 4 (left panels) the basin of attraction of the two-neuron motif changes when ${\rm{\Delta }}{t}^{\ast }$ (blue curve) crosses a certain value of Γ (denoted by arrow). The bistability is between bidirectional (red) and unidirectional (orange) connections (Fig. 4A, left panel), or between decoupled (blue) and unidirectional (orange) states (Fig. 4B, left panel).

### Recurrent Excitatory Networks

**Figure 5:**
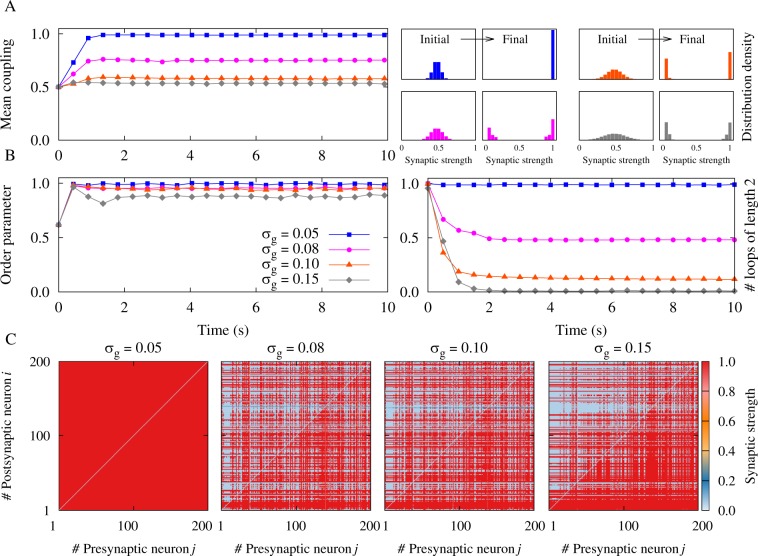
Simulation results for a recurrent excitatory neuronal network in the high-frequency regime with heterogeneous initial synaptic strengths and *ξ* > 0. (**A**) (Left panel) Time course of the mean synaptic strength $\bar{g}$(*t*) for different standard deviations *σ*_*g*_. (Right panel) Initial and final distribution of the synaptic strengths. (**B**) (Left panel) Time course of the order parameter *r*(*t*). Note that the colors indicated in the legend belong to the same *σ*_*g*_ in A and B. (Right panel) Time course of the normalized number of closed loops of length 2 (see Methods), representing the number of bidirectional connections in the network. (**C**) Final coupling matrices for *σ*_*g*_ = 0.05,0.08,0.10,0.15, respectively. Network and STDP parameters are *N* = 200, $\nu =80\,{\rm{H}}{\rm{z}}$, *τ*_d_ = 0.5 ms, *τ*_a_ = 0.3 ms, *A*_+_ = *A*_−_ = 0.005, and *τ*_+_ = *τ*_−_ = 20 ms.

In the two-neuron motif the relative synaptic strength $\Gamma$, given by Eq. (1), determines the final configuration of the connections (see Fig. 4). Small Γ favors more symmetric final structure, and bidirectional connections can emerge if the axonal delays are small. Intriguingly, in large networks the initial distribution of the connection strengths is the key factor determining the emergence of bidirectional connections: In an initially homogeneous network with narrow distribution of the synaptic strengths, bidirectional connections (i.e. two-neuron loops) are more likely to survive in the network, while heterogeneity in the initial synaptic strengths drives the network to an asymmetric final structure with a small number of bidirectional connections. In contrast, so far, the evolution of the network and the emergent structure was attributed only to the mean of the initial synaptic strengths^33,34,38^. In order to study the generalization of our framework to the network level, we took a fixed value of the mean initial synaptic strength and varied the width of the distribution of the initial synaptic strengths by changing the standard deviation *σ*_*g*_ of the distribution. We constructed an initially fully connected network of *N* = 200 excitatory neurons. Synaptic strengths are modified based on the pair-based additive STDP rule given by Eq. (2). Based on the analysis for the case of a two-neuron motif, the dynamics of the network for a given relative delay (difference of axonal and dendritic delays) and firing frequency can be predicted by the initial distribution of the synaptic strengths. Figure 5 shows the results for the recurrent excitatory network with relative delay *ξ* = 0.2 ms, as in the case of short-range synaptic projections, in the high-frequency firing regime. The dendritic delay *τ*_d_ = 0.5 ms is greater than the axonal *τ*_a_ = 0.3 ms. According to Fig. 1G, our analysis of the two-neuron motif predicts a multistability regarding the number of bidirectional and unidirectional connections in the emergent network. Figure 5A, left panel shows the time evolution of the mean synaptic strength, and right panel illustrates the initial and final distribution of the synaptic strengths where the network architecture is dominated by either bidirectional loops (blue distribution) or unidirectional connections (grey distribution). Figure 5B, left panel shows the synchronization degree of the neuronal oscillators and right panel represents the number of two-neuron loops. Figure 5C shows the final coupling matrices for pre- and postsynaptic neurons. For smaller values of the standard deviation (e.g. *σ*_*g*_ = 0.05, 0.08 in Fig. 5C), based on our two-neuron motif analysis, the network is driven to potentiate bidirectional connections. However, for greater values of the standard deviation (e.g. *σ*_*g*_ = 0.10, 0.15 in Fig. 5C), in the emergent structure unidirectional connections have more chance to survive.

**Figure 6:**
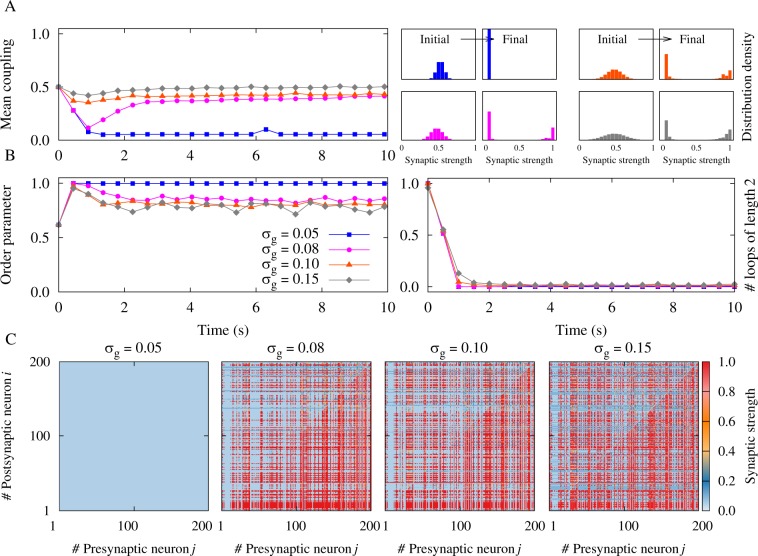
Simulation results for a recurrent excitatory neuronal network in the high-frequency regime with heterogeneous initial synaptic strengths and *ξ* > 0. (**A**) (Left panel) Time course of the mean synaptic strength $\bar{g}$(*t*) for different standard deviations *σ*_*g*_. (Right panel) Initial and final distribution of the synaptic strengths. (**B**) (Left panel) Time course of the order parameter *r*(*t*). (Right panel) Time course of the normalized number of closed loops of length 2, representing the number of bidirectional connections in the network. (**C**) Final coupling matrices for *σ*_*g*_ = 0.05,0.08,0.10,0.15, respectively. Network and STDP parameters are *N* = 200, *ν* = 80 Hz, ${\tau }_{{\rm{d}}}=0.5\,\,{\rm{ms}}$, *τ*_a_ = 1.0 ms, *A*_+_ = *A*_−_ = 0.005, and *τ*_+_ = *τ*_−_ = 20 ms.

For large axonal delays, as in the case of long-range synaptic projections, a similar bistability is observed between unidirectional connections and a disconnected network. Figure 6 shows the results for *ξ* = −0.5 ms. The axonal delay *τ*_a_ = 1.0 ms is greater than the dendritic *τ*_d_ = 0.5 ms. Figure 6A, left panel shows the evolution of the mean synaptic strength in the network, and right panel represents the initial and final distribution of the synaptic strengths where the network structure tends to be decoupled (blue distribution) or dominated by unidirectional connections (grey distribution). Figure 6B, left panel shows the synchronization degree of the neuronal oscillators and right panel represents the number of two-neuron loops. Figure 6C shows the final coupling matrices for pre- and postsynaptic neurons. In the case with small standard deviation of the synaptic strengths (e.g. *σ*_*g*_ = 0.05, 0.08 in Fig. 6C), most of the connections tend to be depressed. In contrast, for wider distributions (e.g. ${\sigma }_{g}=\mathrm{0.10,}\,0.15$ in Fig. 6C), the synapses get unidirectionally potentiated. Note that the stability of the disconnected network in our simulations relies on our choice of the lower hard bound of the synaptic strength, *g*_min_. Assigning a finite value for *g*_min_ stabilizes the network structure at a point where all connections attain the minimum value. Setting the minimum synaptic weight at zero, destabilizes the disconnected network, and one of the synapses builds up to achieve a unidirectional link. Our choice seems reasonable based on the experimental results on STDP^3,5^. The emergence of a stable decoupled state in the phase-locked mode can, e.g., be of importance for the development of stimulation methods for neurological disorders designed to induce long-lasting, sustained therapeutic effects by shifting a neuronal network from phase-locked attractors to more favorable, decoupled attractors^38,54,55^.

**Figure 7:**
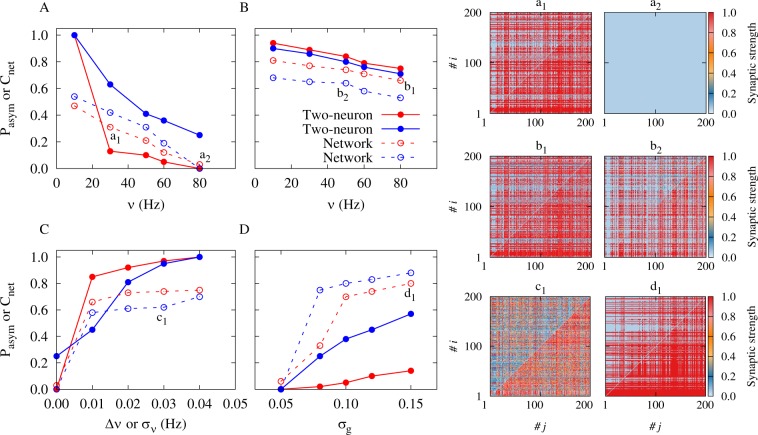
Illustration of the accordance between two-neuron theoretical framework and network simulation results. (**A–D**) The probability *P*_asym_ as a measure of asymmetry in the two-neuron motif (solid) and the asymmetry index ${C}_{{\rm{net}}}$ for the neuronal network (dotted curves) are calculated in the presence of different axonal propagation delays with *τ*_a_ = 0.3 ms (red) and *τ*_a_ = 1.0 ms (blue curves). (**A**) The effect of the firing frequency on *P*_asym_ (*C*_net_) in the two-neuron motif (network simulation) with inhomogeneity in the initial distribution of the synaptic strengths represented by *σ*_*g*_. In the figure *σ*_*g*_ = 0.05. (**B**) Same as A, but in the presence of inhomogeneity in the firing frequencies indicated by Δ*ν* (${\sigma }_{\nu }$) in the two-neuron motif (network simulation). In the figure Δ*ν* = *σ*_*ν*_ = 0.01 Hz. (**C**) The effect of inhomogeneity in the frequencies Δ*ν* (*σ*_*ν*_) on *P*_asym_ (*C*_net_). In the figure *ν* = 80 Hz and *σ*_*g*_ = 0.05. (**D**) Same as C, but for inhomogeneity in the initial distribution of the synaptic strengths *σ*_*g*_. In the figure *ν* = 80 Hz and Δ*ν* = *σ*_*ν*_ = 0.01 Hz. (*a*_1_–d_1_) Samples of final coupling matrix indexed by the number of pre- (*j*) and postsynaptic (*i*) neurons correspond to a_1_–d_1_ markers in A–D, representing the value of the asymmetry index *C*_net_ = 0.31,0.00,0.75,0.64,0.62,0.80 in the simulated network, respectively.

**Figure 8:**
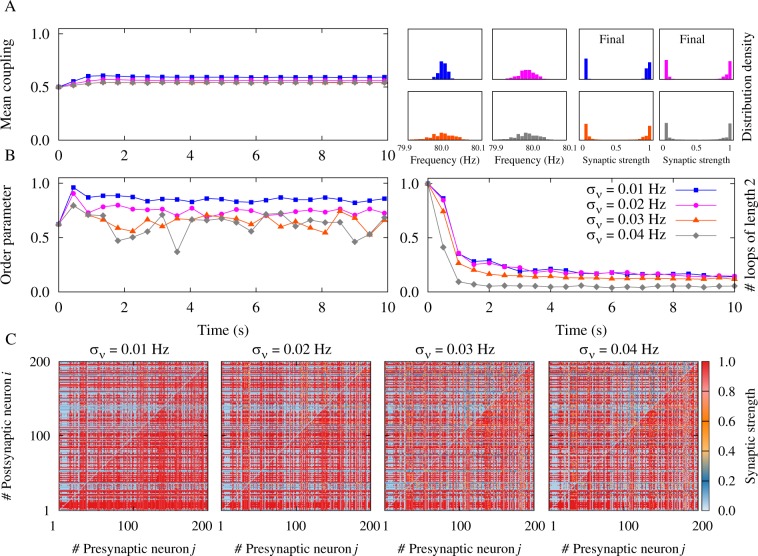
Simulation results for a recurrent excitatory neuronal network in the high-frequency regime with heterogeneous firing frequencies and *ξ* > 0. (**A**) (Left panel) Time course of the mean synaptic strength $\bar{g}$(*t*) for different standard deviations *σ*_*ν*_. (Right panel) Distribution of the firing frequencies and the final synaptic strengths. (**B**) (Left panel) Time course of the order parameter $r(t)$. (Right panel) Time course of the normalized number of closed loops of length 2, representing the number of bidirectional connections in the network. (**C**) Final coupling matrices for *σ*_*ν*_ = 0.01, 0.02, 0.03, 0.04 Hz, respectively. Network and STDP parameters are *N* = 200, $\bar{\nu }$ = 80 Hz, *τ*_d_ = 0.5 ms, ${\tau }_{{\rm{a}}}=0.3\,\,{\rm{ms}}$, *A*_+_ = *A*_−_ = 0.005, and *τ*_+_ = *τ*_−_ = 20 ms.

**Figure 9:**
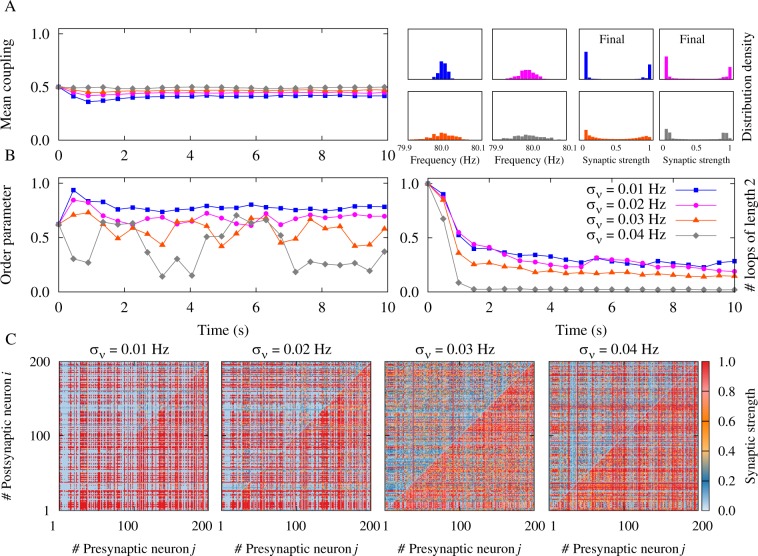
Simulation results for a recurrent excitatory neuronal network in the high-frequency regime with heterogeneous firing frequencies and *ξ* > 0. (**A**) (Left panel) Time course of the mean synaptic strength $\bar{g}$(*t*) for different standard deviations ${\sigma }_{\nu }$. (Right panel) Distribution of the firing frequencies and the final synaptic strengths. (**B**) (Left panel) Time course of the order parameter *r*(*t*). (Right panel) Time course of the normalized number of closed loops of length 2, representing the number of bidirectional connections in the network. (**C**) Final coupling matrices for *σ*_*ν*_ = 0.01, 0.02, 0.03, 0.04 Hz, respectively. Network and STDP parameters are *N* = 200, $\bar{\nu }$ = 80 Hz, *τ*_d_ = 0.5 ms, ${\tau }_{{\rm{a}}}=1.0\,\,{\rm{ms}}$, *A*_+_ = *A*_−_ = 0.005, and *τ*_+_ = *τ*_−_ = 20 ms.

By decreasing the firing frequency and increasing the disparity of the neuronal firing rate the symmetric (bidirectional or decoupled) connections are less likely to survive as revealed by the two-neuron motif analysis (Figs 1 and 3). We extracted the probability (*P*_asym_) of appearance of unidirectional connections in the two-neuron motif, by taking the ratio of the area in the (*g*_21_, *g*_12_) space which leads to unidirectional connections to the total area of the same parameter space. Figure 7A shows that this probability is a decreasing function of frequency. To investigate the impact of this result on the evolution of the network, we defined the network asymmetry index *C*_net_ which quantifies the average asymmetry of the connections between pairs of neurons (see Methods). Expectedly, the network asymmetry index shows a similar decreasing trend which indicates that the number of unidirectional connections in the network decreases with increasing frequency (see Fig. 7A). As shown in Fig. 7B, by increasing the frequency of the oscillations, network asymmetry index decreases, which is consistent with a similar decrease in *P*_asym_ in the two-neuron motif, indicating that at higher frequencies the probability of the appearance of symmetric connections (two-neuron loops or decoupled pairs) increases. Also, based on the results of Fig. 3, a sufficiently pronounced inhomogeneity in the firing rate of the neurons can prevent the formation of strong bidirectional connections. Figures 8 and 9 show the network results in the presence of heterogeneous firing frequencies. Left panels in Figs 8A and 9A represent the mean synaptic strength and right panels illustrate the distribution of the firing frequencies and the final synaptic strengths based on the inhomogeneity in the distribution of the firing rates. Left panels in Figs 8B and 9B show the order parameter of the oscillators, whereas right panels represent the number of two-neuron loops in the network which is suppressed by increasing the inhomogeneity in the distribution of the firing rates. Figures 8C and 9C show the final coupling matrices for pre- and postsynaptic neurons. As shown in Figs 8 and 9, by increasing the standard deviation *σ*_*ν*_ of the distribution of the firing rates, the number of loops in the final structure decreases. This is also illustrated in Fig. 7C where the asymmetry index *C*_net_ increases with increasing *σ*_*ν*_. The probability *P*_asym_ in the two-neuron motif also follows the same ascending trend. Figure 7D shows that by increasing the inhomogeneity in the initial distribution of the synaptic strengths with standard deviation *σ*_*g*_, unidirectional connections with high values of the asymmetry index are more likely to emerge. This increasing trend was also generally predicted by the probability *P*_asym_ in the two-neuron motif based on our theoretical framework.

## Discussion

STDP is conventionally known as a mechanism which generically breaks the structural symmetry of neuronal networks and underlies the formation of feedforward networks^4,19,24,25,56^. This result in recurrent networks contradicts the experimental observations in cortical circuits where bidirectional connections are frequent^26–30^. Bidirectional connections can be retained through alternative versions of conventional STDP: Triplet-based STDP^57^ and STDP with a shifted profile^23^. Triplet-based STDP, which has been designed to conform the dependence of the experimentally observed synaptic changes to the firing rates^58^, can promote bidirectional connections in the high-frequency regime^57^. Pair-based STDP with a rightward shifted profile can also lead to bidirectional connections^23^, if potentiation dominates depression. The imbalance of the STDP profile in favor of potentiation in short spike-pairing times, may result in the retention of bidirectional connections when the neurons are loosely phase-locked^23^. In a more realistic modeling, considering forward and backward propagation delays, we have previously shown^32^ that the widely accepted consequences of conventional STDP, mentioned above, can be challenged. Here we showed that in this framework, the evolution of the bidirectional connections and the symmetry of the emerging structure shows multistability and the degree of heterogeneity in the initial setting of the network determines the final structure of the network.

Experimentally observed pair-based STDP parameters have been shown to be unbalanced: *A*_+_ > *A*_−_ and *τ*_+_ < *τ*_−_^2,3,5^. However, a balanced profile of STDP, i.e. ${A}_{+}\,=\,{A}_{-}$ and *τ*_+_ = *τ*_−_, has widely been used in computational and theoretical studies^4,19–21,23^, since it can provide a suitable substrate for extracting the pure effect of other parameters, e.g. propagation delays, on the dynamics of the system. For example, as noted above, in a potentiation dominated profile of STDP, bidirectional connections can emerge without incorporation of propagation delays in the model^23,31,35^. In the present study we chose a balanced STDP profile to highlight the effects which can be directly attributed to propagation delays.

Previous studies ascribed the final stable connection regime in neuronal networks with synaptic plasticity without propagation delays to the initial mean coupling^33,34^. Here, based on the analysis presented for the two-neuron motif, we focused on the multistability of the network structure due to the disparity of the initial synaptic strengths. Our results show that for every pair of neurons, the final configuration of the synaptic connections follows a bistable dynamics governed by the initial mismatch of the strength of the synaptic connections in both directions, where the initial asymmetry in the structure (difference in the synaptic strengths) specifies the final synaptic configuration. The emerging configurations depend on the difference between dendritic and axonal delays: For larger dendritic delays, the final structures are unidirectional and bidirectional, while for larger axonal delays the two achievable states are unidirectional or fully decoupled neurons. In particular, our results highlight the importance of propagation delays, which are usually neglected in modeling studies of STDP: Without taking into account delays, the bistability cannot emerge, and all initial settings end up with a unidirectional configuration.

Our findings demonstrate the crucial role of the presence and the range of dendritic and axonal propagation delays in regulating the emergent structures of synaptic connections in plastic excitatory neuronal networks. We show that connections with experimentally measured values of short-range delays^41,42^ and gamma band firing rates^58^ might be favorable for strong bidirectional loops or unidirectional connections, depending on the initial distribution of the synaptic strengths (see Fig. 5). In contrast, in the case of long-range connections, a loosely connected network or unidirectional synaptic structures might emerge (see Fig. 6). Therefore, the difference of dendritic and axonal propagation delays is crucial for the selection of the network’s ultimately emerging coupling regimes. In this way, qualitatively distinct connectivity patterns may emerge.

The emergence of multistable coupling regimes can be of interest since such systems can be used in order to construct memory building blocks. Bistability of the synaptic strengths, arising from the positive feedback nature of STDP, results in the emergence of different stable attractor states of the synaptic connections^59^. Switching between different stable attractors enables neuronal networks to maintain and retrieve memories. Bistability of the network dynamics with recurrent connections mediated by STDP has been addressed for stationary^13^ or oscillatory inputs^60^. Bistable dynamics can be characterized by the presence of two extreme stable fixed points of the synaptic strengths at *g*_min_ and *g*_max_, imposed by the hard bound saturation constraint.The choice of the saturation constraints can have significant effects on the final distribution of the synaptic strengths; however, it does not crucially affect the weight dynamics^61^. Therefore, the main results presented in this study, i.e., development of different connectivity patterns can also be qualitatively obtained when soft bounds are imposed on the synaptic strengths.

The dependence of the emergent structure to the firing rate of the neurons was experimentally observed^58^, showing that pairing of spikes with both positive and negative time differences leads to potentiation in the high firing rate regime, which implicitly indicates that bidirectional connections can be promoted in this regime. While previous attempts with pair-based STDP failed to reproduce these results, our study shows that this can be achieved by considering propagation delays without having to consider triplets of spikes in the formulation^32^. Here, we showed that the domain of attraction for each achievable final configuration of the synapses depends on the firing frequency of the neurons. For low firing frequency, the two synapses in the pairs of bidirectional connections evolve in different directions and unidirectional connections are more likely to emerge and survive in the network, as suggested by several studies on STDP^18–23^. However, by increasing the firing frequency, the simultaneous potentiation or depression of the bidirectional connections becomes more probable, so that the measure of the attraction domain for a symmetric configuration in the two dimensional space of the initial synaptic strengths expands. Accordingly, in recurrent neuronal networks the width of the distribution of the synaptic strengths and the firing frequencies determine the emergent structure of the network and, in particular, the number of two-neuron loops in the final structure.

Furthermore, our results show that the symmetric depression of reciprocal connections between neurons in the phase-locked state is possible when favorable combinations of initial synaptic strengths, range of firing rates, and propagation delays are met. We showed that by considering a non-zero lower hard bound *g*_min_, the depression of reciprocal connections can be stabilized in order to construct a loosely connected network, as predicted by our theoretical framework. The possibility of a symmetric stable depression of reciprocal connections in the phase-locked mode may contribute to a further development of brain stimulation techniques that induce an anti-kindling, i.e., an unlearning of abnormally up-regulated synaptic connectivity and, in turn, abnormal synchrony^38^. Coordinated reset (CR) stimulation^62^, a desynchronizing multisite stimulation technique was successfully tested in preclinical^63,64^ and clinical^65–67^ proof of concept studies. However, our approach presented here, may lead to further improvements of brain stimulation techniques causing sustained therapeutic effects.

## Methods

### Pair-Based STDP Model

The neuronal oscillators are subjected to classic pair-based STDP rule where the synaptic strengths are modified based on the learning window function introduced in Eq. (2). The evolution of the synaptic strengths by the pair-based STDP rule can be calculated by taking the average of Eq. (2) over a period of the spiking neuron and smoothing, ${\dot{g}}_{ij}\simeq {\rm{\Delta }}{g}_{ij}/T$ (see Supplementary Fig. S1; for details on the interplay between the temporal scale of the parameters with STDP models see^68^), which is given by:

3 $$\begin{array}{l}{\dot{g}}_{21}=\frac{{A}_{\pm }}{T}[{\rm{sgn}}(\xi +{\rm{\Delta }}t)\,\,\exp \,(-\frac{|\xi +{\rm{\Delta }}t|}{{\tau }_{\pm }})+{\rm{sgn}}\,(T-|\xi +{\rm{\Delta }}t|)\,\exp \,(-\frac{|T-|\xi +{\rm{\Delta }}t||}{{\tau }_{\pm }})],\\ {\dot{g}}_{12}=\frac{{A}_{\pm }}{T}[{\rm{sgn}}(\xi -{\rm{\Delta }}t)\,\,\exp \,(-\frac{|\xi -{\rm{\Delta }}t|}{{\tau }_{\pm }})+{\rm{sgn}}\,(T-|\xi -{\rm{\Delta }}t|)\,\exp \,(-\frac{|T-|\xi -{\rm{\Delta }}t||}{{\tau }_{\pm }})],\end{array}$$

where *A*_+_(*A*_−_) and *τ*_+_(*τ*_−_) are the rate and the effective time window of synaptic potentiation (depression), respectively. *T* is the period of spiking and sgn(Δ*t*) is the sign function. In the entire manuscript the profile of STDP is balanced by setting the parameters to *A*_+_ = *A*_−_ = 0.005, and *τ*_+_ = *τ*_−_ = 20 ms. Synaptic strengths are confined in the interval (*g*_min_, *g*_max_) = [0.05,1]. It should be noted that hard bounds are imposed on the allowed range of synaptic strengths: The synaptic strengths are set to *g*_min_ (*g*_max_) once they cross the lower (upper) limit of their allowed range.

### Phase Reduced Model

Considering that the rate of synaptic change is small, and therefore, the changes in the synaptic strengths are negligible on the fast timescale of the system, the reduced averaged phase model for weakly pulse-coupled neuronal oscillators characterized by intrinsic frequency *ω*_*i*_ = 2*πν*_*i*_ and infinitesimal phase sensitivity *Z*(*φ*) can be written as follows^69,70^:

4 $${\dot{\phi }}_{i}={\omega }_{i}+\frac{1}{2\pi }\sum _{j\mathrm{=1,}j\ne i}^{N}{g}_{ij}\,Z(\psi +{\phi }_{i}-{\phi }_{j}),\,i,j\mathrm{=1,}\,\mathrm{2,}\,\ldots ,\,N,$$

where the neuronal oscillators are coupled via delayed connections of strength *g*_*ij*_ with total delay *τ* = *τ*_d_ + *τ*_a_. *φ*_*i*_ is the phase of the *i*-th oscillator, *ψ* = *ω*_*i*_*τ* is the rescaled delay, and *N* is the number of oscillators. The neurons fire every time their phase passes multiples of 2*π*. In the model, we ignore the synaptic processing time, but the results are not affected by this assumption. Eq. (4) can be written for two coupled type-II phase oscillators with analytical phase response curve (PRC) *Z*(*φ*) = −sin(*φ*):

5 $$\begin{array}{l}{\dot{\phi }}_{1}={\omega }_{1}-\frac{{g}_{12}}{2\pi }\,\sin (\psi +{\phi }_{1}-{\phi }_{2}),\\ {\dot{\phi }}_{2}={\omega }_{2}-\frac{{g}_{21}}{2\pi }\,\sin (\psi +{\phi }_{2}-{\phi }_{1}),\end{array}$$

where subtracting these two relations gives the evolution equation for the relative phase of the two neurons:

6 $$\dot{\chi }=\Omega +\frac{1}{2\pi }[{g}_{12}\,\sin (\psi -\chi )-{g}_{21}\,\sin (\psi +\chi )],$$

where *χ* = *φ*_2_ − *φ*_1_ is the phase lag, and Ω = *ω*_2_ − *ω*_1_. Using the trigonometric identity sin(*x* ± *y*) = sin*x* cos*y* ± cos*x* sin*y*, Eq. (6) can be rearranged to Eq. (1).

### Dynamical Analysis of the Joint Phase Model

In general, the fixed point of the phase lag ${\chi }_{i}^{\ast }$ of the Eq. (6) for type-I neuronal oscillator with analytical PRC function *Z*(*ψ* ± *χ*) = 1 − cos(*ψ* ± *χ*), is given by:

7 $$\begin{array}{l}{\chi }_{1}^{\ast }={\tan }^{-1}(-\frac{2\pi {\rm{\Omega }}+({g}_{21}-{g}_{12})(\cos \,\psi \,\cos \,{\chi }_{1}^{\ast }-\mathrm{1)}}{({g}_{21}+{g}_{12})(\sin \,\psi \,\cos \,{\chi }_{1}^{\ast })}),\\ {\chi }_{2}^{\ast }=\pi -{\chi }_{1}^{\ast },\end{array}$$

where ${\chi }_{1}^{\ast }$ is the inphase firing solution and ${\chi }_{2}^{\ast }$ belongs to antiphase state. Given the synaptic strengths, only one of these fixed points are stable in a given Ω and delay time *ψ*. The fixed points of type-II neuronal oscillator with analytical PRC function $Z(\psi \pm \chi )=-\,\sin (\psi \pm \chi )$ can be derived similarly:

8 $$\begin{array}{l}{\chi }_{1}^{\ast }={\tan }^{-1}(\frac{2\pi {\rm{\Omega }}-({g}_{21}-{g}_{12})\sin \,\psi \,\cos \,{\chi }_{1}^{\ast }}{({g}_{21}+{g}_{12})(\cos \,\psi \,\cos \,{\chi }_{1}^{\ast })}),\\ {\chi }_{2}^{\ast }=\pi -{\chi }_{1}^{\ast }\mathrm{.}\end{array}$$

Eqs (7) and (8) show that the fixed points of both type-I and type-II neuronal oscillations are self-consistent in the presence of intrinsic frequency mismatch Ω between the two oscillators. In this case, the stable fixed point ${\chi }_{i}^{\ast }$ is simply where the two ${y}_{1}={\chi }_{i}^{\ast }$ and ${y}_{2}={\tan }^{-1}(f(\psi ,{\chi }_{i}^{\ast }))$ curves intersect. The other approach is to solve the equation ${\chi }_{i}^{\ast }-{\tan }^{-1}(f(\psi ,{\chi }_{i}^{\ast }))=0$ numerically using any root-finding scheme. However, without loss of generality, one can assume that the intrinsic frequency mismatch between the two oscillators is negligible, i.e. ${\rm{\Omega }}\simeq 0$. Ignoring Ω, the type-I fixed point is still self-consistent, but the type-II fixed point can be simplified to:

9 $$\begin{array}{l}{\chi }_{1}^{\ast }={\tan }^{-1}(-\frac{({g}_{21}-{g}_{12})\tan \,\psi }{{g}_{21}+{g}_{12}})\equiv {\tan }^{-1}({\rm{\Gamma }}\,\tan \,\psi ),\,{\rm{\Gamma }}=|\frac{{g}_{12}-{g}_{21}}{{g}_{12}+{g}_{21}}|,\\ {\chi }_{2}^{\ast }=\pi -{\chi }_{1}^{\ast },\end{array}$$

where ${\rm{\Gamma }}$ is a positive expression that reflects the relative synaptic strength.

### Network Model

A fully connected recurrent excitatory network with *N* = 200 type-II neuronal phase oscillators were simulated in the high-frequency regime with *ν* = 80 Hz. In the network simulation, the initial mean coupling is fixed at $\bar{g}$(0) = 0.5. The elements of the adjacency matrix **A** are multiplied by the corresponding array in the synaptic strength matrix **G** in order to establish a weighted adjacency matrix **A**_**G**_. Note that the main diagonal arrays of the weighted adjacency matrix are zero since there is no self-loop in the network. Phase oscillators obey Eq. (1) and the excitatory synapses are modified by the pair-based STDP profile according to Eq. (2). The phases of the oscillators are initially uniformly distributed between 0 and *π*. The dendritic propagation delay is fixed at *τ*_d_ = 0.5 ms. STDP parameters are *A*_+_ = *A*_−_ = 0.005, and *τ*_+_ = *τ*_−_ = 20 ms. We also define an order parameter *r*(*t*) for the network of phase oscillators ranging between 0 and 1 that measures the degree to which the system is synchronized^71^:

10 $$r{e}^{i{\rm{\Psi }}}={N}^{-1}\sum _{j\mathrm{=1}}^{N}{e}^{i{\phi }_{j}},$$

where Ψ(*t*) is the average phase and *N* is the number of neuronal oscillators.

### Counting Loops

A bidirectional connection corresponds to a closed loop of length 2 in a network of neurons. In order to measure the number of such closed loops, a directed graph is constructed. Transformation of the synaptic strength matrix G, into a directed graph is performed by considering a threshold *h* = 0.2. Assuming that there are no self-loops, i.e. ${g}_{ii}=0$, the corresponding value in the graph adjacency matrix **M** of the resultant directed graph is assigned to 1 when the synaptic strength is greater than *h*, and zero otherwise. The number of closed loops of length 2 in the graph adjacency matrix **M** is then^21,23^:

11 $${L}_{2}=\frac{1}{2}{\rm{Tr}}({{\bf{M}}}^{2}),$$

where Tr denotes the matrix trace. In order to perform a better comparison, *L*_2_ is normalized to the total number of possible loops of the same length i.e. *N*(*N* − 1)/2, ignoring self-loops, where *N* denotes the number of the neuronal phase oscillators or nodes in the network. Therefore, the resulted *L*_2_ is a normalized number between 0 and 1.

### Asymmetry Index

To quantify the proportion of unidirectional connections and discriminate asymmetric modification of the synapses from symmetric changes (either symmetric potentiation or symmetric depression), we used another measure which has been introduced by Bayati and Valizadeh^17^. We first defined the synaptic cost of the network as the sum of all synaptic strengths in the weighted adjacency matrix ${{\bf{A}}}_{{\bf{G}}}$, i.e. $S={\sum }_{i,j}{a}_{ij}{g}_{ij}$, where *a*_*ij*_ and *g*_*ij*_ are the arrays of adjacency **A** and synaptic strength **G** matrices, respectively. The asymmetry level of the connections between two neurons can be measured by calculating the quantity *C*_*ij*_ = −*C*_*ji*_ = *a*_*ij*_ *g*_*ij*_ − *a*_*ji*_ *g*_*ji*_. The asymmetry index of the network can then be defined as ${C}_{{\rm{net}}}\mathrm{=(1/}S){\sum }_{i > j}|{C}_{ij}|$. By definition, the network asymmetry index *C*_net_ is scaled in the range [0,1]. In the case of a fully symmetric network, *C*_net_ = 0, whereas in a fully asymmetric network, *C*_net_ = 1.

## Electronic supplementary material

Supplementary Information

## Data Availability

All data generated or analysed during this study are included in this published article (and its Supplementary Information files).

## References

[1] Gerstner W, Kempter R, van Hemmen JL, Wagner H (1996). A neuronal learning rule for sub-millisecond temporal coding. Nature.

[2] Markram H, Lübke J, Frotscher M, Sakmann B (1997). Regulation of synaptic efficacy by coincidence of postsynaptic aps and epsps. Science.

[3] Bi GQ, Poo MM (1998). Synaptic modifications in cultured hippocampal neurons: dependence on spike timing, synaptic strength, and postsynaptic cell type. Journal of Neuroscience.

[4] Song S, Miller KD, Abbott LF (2000). Competitive hebbian learning through spike-timing-dependent synaptic plasticity. Nature Neuroscience.

[5] Bi GQ, Poo MM (2001). Synaptic modification by correlated activity: Hebb’s postulate revisited. Annual Review of Neuroscience.

[6] Kempter R, Gerstner W, van Hemmen JL (2001). Intrinsic stabilization of output rates by spike-based hebbian learning. Neural Computation.

[7] Gütig R, Aharonov R, Rotter S, Sompolinsky H (2003). Learning input correlations through nonlinear temporally asymmetric hebbian plasticity. Journal of Neuroscience.

[8] Izhikevich EM, Gally JA, Edelman GM (2004). Spike-timing dynamics of neuronal groups. Cerebral Cortex.

[9] Morrison A, Aertsen A, Diesmann M (2007). Spike-timing-dependent plasticity in balanced random networks. Neural Computation.

[10] Gilson M, Burkitt AN, Grayden DB, Thomas DA, van Hemmen JL (2009). Emergence of network structure due to spike-timing-dependent plasticity in recurrent neuronal networks. i. input selectivity–strengthening correlated input pathways. Biological Cybernetics.

[11] Gilson M, Burkitt AN, Grayden DB, Thomas DA, van Hemmen JL (2009). Emergence of network structure due to spike-timing-dependent plasticity in recurrent neuronal networks. ii. input selectivity–symmetry breaking. Biological Cybernetics.

[12] Gilson M, Burkitt AN, Grayden DB, Thomas DA, van Hemmen JL (2009). Emergence of network structure due to spike-timing-dependent plasticity in recurrent neuronal networks. iii. partially connected neurons driven by spontaneous activity. Biological Cybernetics.

[13] Gilson M, Burkitt AN, Grayden DB, Thomas DA, van Hemmen JL (2009). Emergence of network structure due to spike-timing-dependent plasticity in recurrent neuronal networks. iv. Biological Cybernetics.

[14] Gilson M, Burkitt AN, Grayden DB, Thomas DA, van Hemmen JL (2010). Emergence of network structure due to spike-timing-dependent plasticity in recurrent neuronal networks. v. self-organization schemes and weight dependence. Biological Cybernetics.

[15] Gilson M, Burkitt A, van Hemmen LJ (2010). Stdp in recurrent neuronal networks. Frontiers in Computational Neuroscience.

[16] Mikkelsen K, Imparato A, Torcini A (2013). Emergence of slow collective oscillations in neural networks with spike-timing dependent plasticity. Physical Review Letters.

[17] Bayati M, Valizadeh A (2012). Effect of synaptic plasticity on the structure and dynamics of disordered networks of coupled neurons. Physical Review E.

[18] Abbott LF, Nelson SB (2000). Synaptic plasticity: taming the beast. Nature Neuroscience.

[19] Song S, Abbott LF (2001). Cortical development and remapping through spike timing-dependent plasticity. Neuron.

[20] Lubenov EV, Siapas AG (2008). Decoupling through synchrony in neuronal circuits with propagation delays. Neuron.

[21] Kozloski J, Cecchi GA (2010). A theory of loop formation and elimination by spike timing-dependent plasticity. Frontiers in Neural Circuits.

[22] Knoblauch A, Hauser F, Gewaltig M-O, Körner E, Palm G (2012). Does spike-timing-dependent synaptic plasticity couple or decouple neurons firing in synchrony?. Frontiers in Computational Neuroscience.

[23] Babadi B, Abbott LF (2013). Pairwise analysis can account for network structures arising from spike-timing dependent plasticity. PLoS Computational Biology.

[24] Karbowski J, Ermentrout GB (2002). Synchrony arising from a balanced synaptic plasticity in a network of heterogeneous neural oscillators. Physical Review E.

[25] Masuda N, Kori H (2007). Formation of feedforward networks and frequency synchrony by spike-timing-dependent plasticity. Journal of Computational Neuroscience.

[26] Goldman-Rakic PS (1987). Development of cortical circuitry and cognitive function. Child Development.

[27] Markram H, Lübke J, Frotscher M, Roth A, Sakmann B (1997). Physiology and anatomy of synaptic connections between thick tufted pyramidal neurones in the developing rat neocortex. The Journal of Physiology.

[28] Song S, Sjöström PJ, Reigl M, Nelson S, Chklovskii DB (2005). Highly nonrandom features of synaptic connectivity in local cortical circuits. PLoS Biology.

[29] Morishima M, Kawaguchi Y (2006). Recurrent connection patterns of corticostriatal pyramidal cells in frontal cortex. Journal of Neuroscience.

[30] Douglas RJ, Martin KA (2007). Recurrent neuronal circuits in the neocortex. Current Biology.

[31] Lücken L, Popovych OV, Tass PA, Yanchuk S (2016). Noise-enhanced coupling between two oscillators with long-term plasticity. Physical Review E.

[32] Madadi Asl M, Valizadeh A, Tass PA (2017). Dendritic and axonal propagation delays determine emergent structures of neuronal networks with plastic synapses. Scientific Reports.

[33] Maistrenko YL, Lysyansky B, Hauptmann C, Burylko O, Tass PA (2007). Multistability in the kuramoto model with synaptic plasticity. Physical Review E.

[34] Popovych OV, Tass PA (2012). Desynchronizing electrical and sensory coordinated reset neuromodulation. Frontiers in Human Neuroscience.

[35] Popovych OV, Yanchuk S, Tass PA (2013). Self-organized noise resistance of oscillatory neural networks with spike timing-dependent plasticity. Scientific Reports.

[36] Popovych OV, Tass PA (2014). Control of abnormal synchronization in neurological disorders. Frontiers in Neurology.

[37] Tass PA (2017). Vibrotactile coordinated reset stimulation for the treatment of neurological diseases: Concepts and device specifications. Cureus.

[38] Tass PA, Majtanik M (2006). Long-term anti-kindling effects of desynchronizing brain stimulation: a theoretical study. Biological Cybernetics.

[39] Agmon-Snir H, Segev I (1993). Signal delay and input synchronization in passive dendritic structures. Journal of Neurophysiology.

[40] Schierwagen A, Claus C (2001). Dendritic morphology and signal delay in superior colliculus neurons. Neurocomputing.

[41] Cleland B, Levick W, Morstyn R, Wagner H (1976). Lateral geniculate relay of slowly conducting retinal afferents to cat visual cortex. The Journal of Physiology.

[42] Swadlow HA, Weyand TG (1987). Corticogeniculate neurons, corticotectal neurons, and suspected interneurons in visual cortex of awake rabbits: receptive-field properties, axonal properties, and effects of eeg arousal. Journal of Neurophysiology.

[43] Swadlow HA (1990). Efferent neurons and suspected interneurons in s-1 forelimb representation of the awake rabbit: receptive fields and axonal properties. Journal of Neurophysiology.

[44] Ernst U, Pawelzik K, Geisel T (1995). Synchronization induced by temporal delays in pulse-coupled oscillators. Physical Review Letters.

[45] Ermentrout B, Pascal M, Gutkin B (2001). The effects of spike frequency adaptation and negative feedback on the synchronization of neural oscillators. Neural Computation.

[46] Sadeghi S, Valizadeh A (2014). Synchronization of delayed coupled neurons in presence of inhomogeneity. Journal of Computational Neuroscience.

[47] Esfahani ZG, Gollo LL, Valizadeh A (2016). Stimulus-dependent synchronization in delayed-coupled neuronal networks. Scientific Reports.

[48] Ermentrout B (1996). Type i membranes, phase resetting curves, and synchrony. Neural Computation.

[49] Achuthan S, Canavier CC (2009). Phase-resetting curves determine synchronization, phase locking, and clustering in networks of neural oscillators. Journal of Neuroscience.

[50] Câteau H, Kitano K, Fukai T (2008). Interplay between a phase response curve and spike-timing-dependent plasticity leading to wireless clustering. Physical Review E.

[51] Kempter R, Gerstner W, Van Hemmen JL (1999). Hebbian learning and spiking neurons. Physical Review E.

[52] Aoki T, Aoyagi T (2009). Co-evolution of phases and connection strengths in a network of phase oscillators. Physical Review Letters.

[53] Pariz A (2018). High frequency neurons determine effective connectivity in neuronal networks. NeuroImage.

[54] Zeitler M, Tass PA (2015). Augmented brain function by coordinated reset stimulation with slowly varying sequences. Frontiers in Systems Neuroscience.

[55] Zeitler M, Tass PA (2016). Anti-kindling induced by two-stage coordinated reset stimulation with weak onset intensity. Frontiers in Computational Neuroscience.

[56] Bayati M, Valizadeh A, Abbassian A, Cheng S (2015). Self-organization of synchronous activity propagation in neuronal networks driven by local excitation. Frontiers in Computational Neuroscience.

[57] Pfister JP, Gerstner W (2006). Triplets of spikes in a model of spike timing-dependent plasticity. Journal of Neuroscience.

[58] Sjöström PJ, Turrigiano GG, Nelson SB (2001). Rate, timing, and cooperativity jointly determine cortical synaptic plasticity. Neuron.

[59] Chaudhuri R, Fiete I (2016). Computational principles of memory. Nature Neuroscience.

[60] Pfister JP, Tass P (2010). Stdp in oscillatory recurrent networks: theoretical conditions for desynchronization and applications to deep brain stimulation. Frontiers in Computational Neuroscience.

[61] Morrison A, Diesmann M, Gerstner W (2008). Phenomenological models of synaptic plasticity based on spike timing. Biological Cybernetics.

[62] Tass PA (2003). A model of desynchronizing deep brain stimulation with a demand-controlled coordinated reset of neural subpopulations. Biological Cybernetics.

[63] Tass PA (2012). Coordinated reset has sustained aftereffects in parkinsonian monkeys. Annals of Neurology.

[64] Wang J (2016). Coordinated reset deep brain stimulation of subthalamic nucleus produces long-lasting, dose-dependent motor improvements in the 1-methyl-4-phenyl-1, 2, 3, 6-tetrahydropyridine non-human primate model of parkinsonism. Brain Stimulation: Basic, Translational, and Clinical Research in Neuromodulation.

[65] Tass PA, Adamchic I, Freund HJ, von Stackelberg T, Hauptmann C (2012). Counteracting tinnitus by acoustic coordinated reset neuromodulation. Restorative Neurology and Neuroscience.

[66] Adamchic I (2014). Coordinated reset neuromodulation for parkinson’s disease: Proof-of-concept study. Movement Disorders.

[67] Syrkin-Nikolau J (2018). Coordinated reset vibrotactile stimulation shows prolonged improvement in parkinson’s disease. Movement Disorders.

[68] Gilson M, Bürck M, Burkitt AN, van Hemmen JL (2012). Frequency selectivity emerging from spike-timing-dependent plasticity. Neural Computation.

[69] Izhikevich EM (1999). Weakly pulse-coupled oscillators, fm interactions, synchronization, and oscillatory associative memory. IEEE Transactions on Neural Networks.

[70] Hoppensteadt, F. C. & Izhikevich, E. M. *Weakly connected neural networks* (Springer Science & Business Media, 1997).

[71] Kuramoto, Y. *Chemical oscillations, waves, and turbulence* (Springer Science & Business Media, 1984).

