# Supplementary material for: Delay-Induced Multistability and Loop Formation in Neuronal Networks with Spike-Timing-Dependent Plasticity
Source: Sci Rep. 2018 Aug 13;8:12068. doi: 10.1038/s41598-018-30565-9 (PMC6089910; doi:10.1038/s41598-018-30565-9)
Supplement: Supplementary file 1 — Supplementary Information [file 41598_2018_30565_MOESM1_ESM.pdf]

Supplementary Information for: Delay-Induced Multistability and Loop Formation in Neuronal Networks with Spike-Timing-Dependent Plasticity

Mojtaba Madadi Asl, Alireza Valizadeh, and Peter A. Tass

Evolution of the synaptic strengths by pair-based STDP

In our formalism in Eq. (3), we assumed that the neurons spike periodically and are strictly phase-locked so that the distribution of spike timing differences has a double delta distribution with two peaks at Δt and $T-\Delta t$. We then calculated the net change over a period Δg_{ij} as the difference between two successive potentiation and depression events, and assumed that the synaptic strength evolves smoothly over a period by the rate $\Delta g_{ij}/T$ (see Fig. **S1**, bottom). Extending these considerations to the case of a network, specifically with a narrow distribution for the spike timing differences in our formalism (which is correct for a more general case when the neurons are not strictly phase-locked and the phase differences have a jitter due to noise or network effects), the results are qualitatively the same. The dependence of the results on the spiking period T and, consequently, on the frequency of the oscillation can be seen when the period is comparable with other timescales of the system such as that of the plasticity window. However, by letting T be much larger than other timescales of the system, the dependence on the frequency of the oscillation disappears. For details on the interplay between the temporal scale of the parameters with STDP models see [1]. As an alternative and basically similar approach, we can begin with the formula $\dot{g}_{ij} = \int K(s)S(t-s)ds$, with $K(s)$ being the STDP kernel and $S(t)$ being the joint spike distribution, for updating the synaptic strengths. A double delta distribution for $S(t)$ which is a consequence of complete phase-locking and periodic spiking of the neurons results in Eq. (3) after averaging over one period and smoothing as noted above.

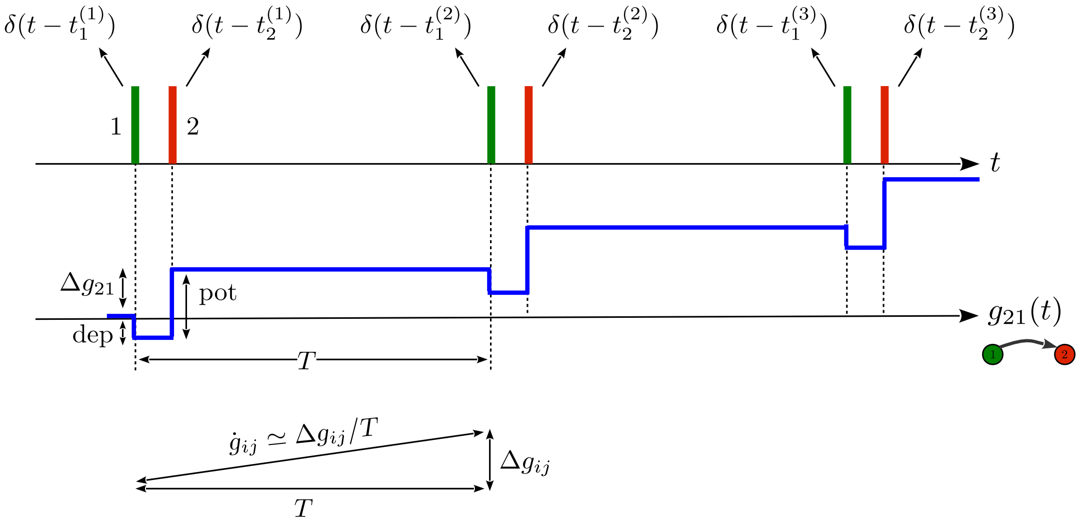

Figure S1. **Schematic evolution of the synaptic strengths.** (Top axis) The spike trains of neurons 1 (green) and 2 (red) are shown as delta functions, $S_1(t) = \sum_f \delta(t-t_1^{(f)})$ and $S_2(t) = \sum_f \delta(t-t_2^{(f)})$, where $t_i^{(f)}$ is the f-th firing of the neuron i. (Bottom axis) Evolution of the strength of the synapse $1 \longrightarrow 2$ is shown ($g_{21}(t)$, blue) in a setting that leads to the potentiation of the synapse. In each period T, the change in the synaptic strength consists of a potentiation and a depression term which compete to determine the net change Δg_{21}. The evolution of the synaptic strength $\dot{g}_{21}$ can be then considered as the slope of this change, $\dot{g}_{21} \simeq \Delta g_{21}/T$.

References

[1] Gilson, M., Bürck, M., Burkitt, A. N., & van Hemmen, J. L. Frequency selectivity emerging from spike-timing-dependent plasticity. Neural Computation, 24(9), 2251-2279 (2012).
